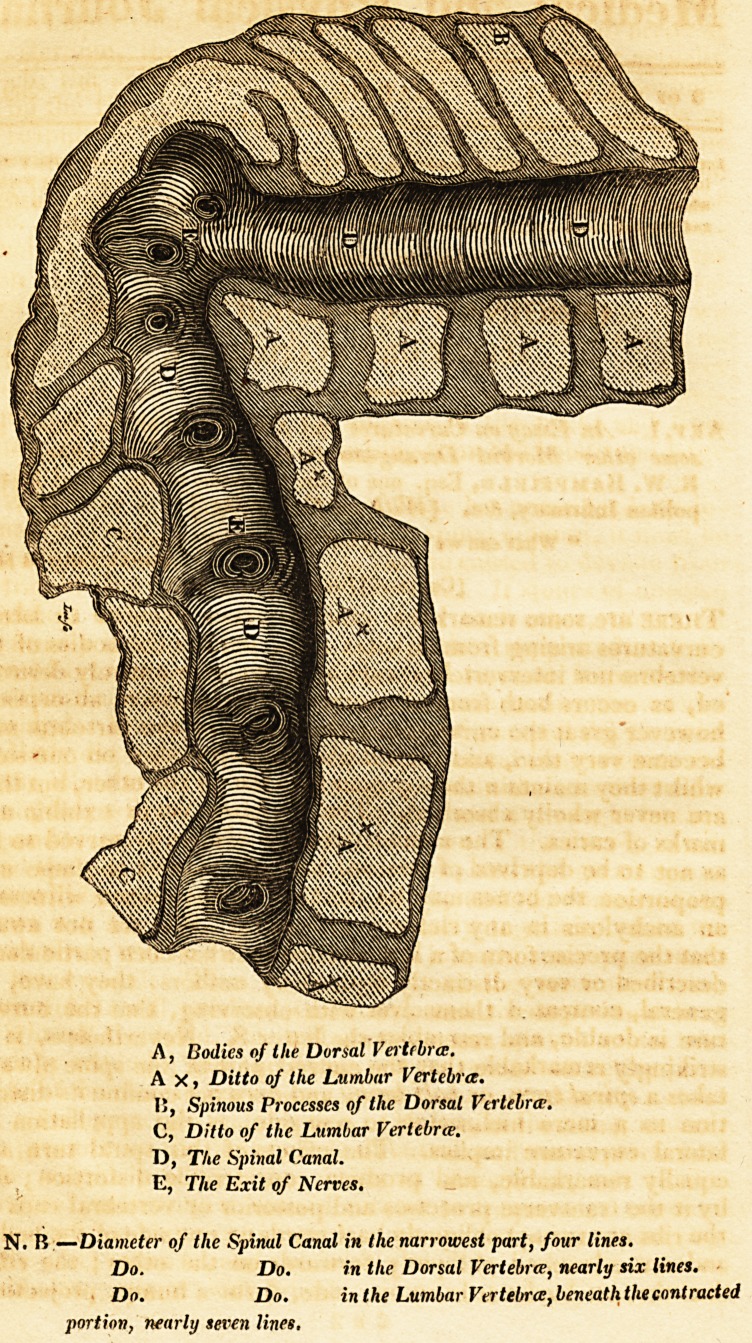# An Essay on Curvatures and Distortions of the Spine, and Some Other Morbid Derangements to Which It Is Subject

**Published:** 1823-03

**Authors:** R. W. Bampfield

**Affiliations:** one of the Surgeons to the Royal Metropolitan Infirmary, &c.


A, *Bodies of the Dorsal Vertebræ.*

A ×, *Ditto of the Lumbar Vertebræ.*

B, *Spinous Processes of the Dorsal Vertebræ.*

C, *Ditto of the Lumbar Vertebræ.*

D, *The Spinal Canal.*

E, *The Exit of Nerves.*

N. B.—*Diameter of the Spinal Canal in the narrowest part, four lines.*
*Do. Do. in the Dorsal Vertebræ, nearly six lines.*
*Do. Do. in the Lumbar Vertebræ, beneath the contracted portion, nearly seven lines.*

THE LONDON

### Medical and Physical Journal.

3 OF VOL. XLIX.] MARCH, 1823. [No. 289.

For many fortunate discoveries in medicine, and for the detection of numerous errors, the world is indebted to the rapid circulation of Monthly Journals; and there never existed any work, to which the Faculty, in Europe and America, were under deeper obligations, than to the Medical and Physical Journal of London, now forming a long, but an invaluable, series.—RUSH.

### ORIGINAL COMMUNICATIONS,

### SELECT OBSERVATIONS, &c.

ART. I.—*An Essay on Curvatures and Distortions of the Spine, and some other Morbid Derangements to which it is subject.* By R. W. BAMPFIELD, Esq. one of the Surgeons to the Royal Metropolitan Infirmary, &c. [*With an Engraving.*]

"What can we reason, but from what we know?"

POPE'S *Essay on Man.*

[Continued from page 77.]

THERE are some remarkable circumstances peculiar to lateral curvatures arising from rickets: thus, neither the bodies of the vertebræ nor intervertebral cartilages are ever entirely destroyed, as occurs both from ulcerative and progressive absorption, however great the curvatures; the bodies of the vertebræ may become very thin, and shaped like a sharp wedge on one side, whilst they maintain their proper breadth on the other, but they are never wholly absorbed. The vertebræ never exhibit any marks of caries. The articulations are always preserved so far as not to be deprived of motion, however altered in shape and proportion the bones may be, so that we have never witnessed an anchylosis in any ricketty curvature. We are not aware that the precise form of a lateral curvature has been particularly described or very distinctly noticed by authors: they have, in general, contented themselves with observing, that the curvature is double, and resembles the letter S. Nevertheless, it is strikingly remarkable that the curved part of the spine always takes a *spiral turn*, or *half turn*, and does not confine its distortion to a mere inclination to one side, as the appellation of lateral curvature implies. The effects of this spiral turn are equally remarkable, and produce considerable distortion; for by it the transverse processes and posterior or vertebral ends of the ribs are turned obliquely backwards on one side of the body, and are advanced obliquely forwards on the other; the ribs, thus thrown backwards on one side, form a hump, projecting more or less than an inch posteriorly to the spinal column, and, in the longer course or turn they are obliged to take to arrive at the sternum, they sometimes closely approximate, or actually touch, the anterior portions of the vertebræ. As the scapulæ lie on the ribs, and the clavicle is articulated to the scapula at its scapular extremity, these bones and the shoulder must follow the direction of the ribs, and be carried back on the side the ribs project backwards, and be turned forwards on the other side. In some cases, it has appeared to me that the lower extremities on the side advancing forwards are somewhat shorter than on the other; but whether this be caused by the spiral turn of the spine, or be an independent effect of rachitis, we cannot at present decide with certainty. Where there is no ricketty diathesis, the cervical vertebræ are subject to direct lateral curvatures from the pressure of tumors on, and long-continued contractions of, the muscles of the neck, and from untoward position of the neck, arising from bad habits and large heads.

Whenever a ricketty growth of bone has occasioned an inequality of any of the vertebræ of the spinal column, it must be determined from its natural axis, and be caused to deviate from a straight line to a curve. To illustrate. If stones of unequal thickness be successively disposed over each other, they must eventually incline to one side, and, if then supported, will be formed into an arc, such as is observed to result from the disposition made of cuneiform stones in constructing the arch of a bridge. If several elastic substances of unequal thickness be similarly disposed, with joints and mechanical contrivances (as in the joints of the spinal column,) to retain them in their situation, they will necessarily form an arc; and if a weight be then appended or superadded to the extreme of the curve, it will bend the pile so as to increase the curve, and the greater pressure will be sustained by its inner circle, and the surface of the joints will be there pressed very closely together, while the outer circle will be enlarged, and the joints in some parts more or less opened. Thus the inequality of the vertebræ arising from rickets will produce curvature, and the superincumbent weight of the head, &c. will increase that curve: so far the analogy extends; but the living powers possess an active principle of increase of curvature, which inanimate matter is devoid of. This principle is absorption. Hence, in the living matter, the pressure on the inner circle of curvature from superincumbent weight produces the action of absorption, and if the vertebræ pressed upon remain subject to frequent or constant pressure from superincumbent weight, by the patient indulging in the erect attitude, and continuing to sit up and walk about as usual, the pressure will promote continued absorption, which will conduce much more to the increase of the curvature, than the pristine or any successive inequality in the growth of bone which has its natural limits. To convey more precise notions of the effects of unequal growth of bone in the spinal column, and of the consequent absorption, we will take the aid an accurate admeasurement of the affected vertebræ affords. In a specimen, in Mr. Brookes's museum, of double lateral curvature exhibiting the spiral turn, in which there is not the slightest appearance of caries, the upper curvature more particularly involves the third, fourth, and fifth dorsal, and the lower the tenth, eleventh, and twelfth; all the dorsal vertebræ are either bent or twisted from their proper situation, and deviate more or less from their natural proportions. In this specimen, the inner line of curvature of the tenth, eleventh, and twelfth dorsal vertebræ admeasures only one inch four lines, while the outer line measures three inches three lines. The breadth of the right side of the eleventh dorsal vertebra is only three lines, whilst the breadth of the left is one inch. The breadth of the right side of the tenth and twelfth dorsal vertebræ is half an inch, of the left side one inch. In another specimen, the breadth of the eighth dorsal vertebra on the right side is only two lines, while on the opposite side it is one inch, and three other vertebræ are proportionably diminished.

The speculative pathologist might find some curious exercise for his mind in endeavouring to account for the different effects on the spinal column of the same action—absorption, produced by the same cause—pressure, in the ricketty and non-ricketty distortions of the spine. In the former, the bodies become gradually diminished in thickness and cuneiform, but not carious or entirely absorbed, whilst the intervertebral substances remain entire, and preserve the joints and the vertebræ distinct. In some cases of the latter, the bodies, cartilages, ligaments, &c. will eventually become quite absorbed. Can it be attributed to a different disposition in the action of the absorbents, or to different degrees of hardness in the texture of the bones?* or can it arise from the difference in the power or force of the pressure, which in the ricketty distortion is diminished by the spiral turn, and by the double curvature presenting two points of pressure, which necessarily relieve each other, and divide the compressing power of the superincumbent weight?

Incurvations of the spine are less frequent than excurvations, lateral curvatures, or angular projections, and do not describe so large a curve as the two former; at least, such is the conclusion from our present experience. The difference in the

* We have not noticed the faulty action of the depositing arteries in ricketty bones; but, as the effect seems to be to render them more susceptible of absorption, we perhaps should have attached more importance to them.

anatomical structure of the posterior and anterior portions of the vertebræ will account for the circumstance of incurvations seldom obtaining so extensive a curvature as the excurvations, and will also explain why incurvations occur more frequently in the cervical and lumbar vertebræ than in the dorsal; for the spinous processes in the latter oppose a greater resistance by their closer apposition than in the former, where they are more distantly separated. In their natural state, the intervertebral substances yield readily to compression, and allow the spinal column to bend forwards much more than the oblique and spinous processes do backwards, although the power of youthful habit and training, exemplified in tumblers, can accomplish much in increasing the ability of bending backwards, without displacing any of the vertebræ. Incurvations of the lumbar vertebræ are frequently accompanied with lumbar and psoas abscesses, with which, in such cases, they appear to be connected as cause and effect, and reciprocally produce each other. Thus the pus contained in the cyst of a psoas abscess, or within the parietes of a lumbar abscess, may by its pressure produce absorption of the vertebræ, or occasion caries of bone and ulcerative absorption of both bone and cartilage; and caries of bone, or ulcerative disease of the vertebral joints, may occasion the accumulation of pus forming psoas abscess. In Mr. Brookes's museum, there is a specimen of a complete excavation of the body of the second lumbar vertebra by the pressure of the cyst of a psoas abscess on the right side. It may be remarked from this specimen, in illustration of what has been formerly said relative to the mode by which curvatures are produced, that the lower horizontal surface of the second lumbar vertebra has been destroyed by caries, except at two anterior points, by which, as by pillars, the vertebral column has been supported, and the curvature prevented. It should be observed, that we have witnessed more cases of psoas abscess connected or combined with excurvations or curvatures outwards, than we have with incurvations. Incurvations are also occasioned by an increased growth of the anterior portions of the bodies of the vertebræ, and by the permanent contraction of the flexor muscles of the thigh situated within the abdomen.

It has been stated that tumors, growing and enlarging in the vicinity of the spine, produce different species of curvature: thus, tumors on the neck frequently occasion lateral curvatures of the cervical vertebræ. It is, however, proper to mention that they sometimes effectually oppose the formation of curvature, even when the horizontal and perpendicular portions of the bodies of several vertebræ and intervertebral substances, as well as their ligamentum anticum commune, are, for the most part, destroyed by ulcerative or progressive absorption, occasioned by their pressure: in such cases, the tumors have filled up the space the bodies of the vertebræ occupied, and have thus prevented this part of the column from falling out of its spinal line.* It is possible that great natural efforts are instinctively made, in such instances, to maintain the erect attitude of the diseased parts, in order to preserve the sufferer from the pain which inclining any superincumbent weight on the tumor would necessarily create; for nature always endeavours to avoid the motions and positions which bring pain in their train. It may also be presumed that such patients generally observe the horizontal, or that posture which affords most ease.

It has been already laid down as a general position, that the absorption which takes place in curvatures of the spine must be in the ratio of the compressing power, and of the liability to absorption of the parts compressed. Thus it is, adult patients, or the parents of children, so frequently represent that, after the curvature has attained some extent, it has increased very rapidly. This may be partly explained by a principle in mechanics. If a weight be affixed to the upper extremity of an elastic column, curved to the degree of one, and the same weight be placed upon the upper extremity of another, curved to the degree of three or four,—in the latter case, the superincumbent weight will tend to increase the bending of the column with a greater force and effect than in the former: hence, the more extensive the curvature, the greater will be the compressing power of the superincumbent weight, and the quicker the absorption that is established and going on. From the appearance of the remains of some parts of the bodies of the vertebræ, it may be reasonably inferred that the action of the absorbents continues in the vertebra that has been exposed to compression for a short time after pressure has been removed. These and some other points relative to absorption of the vertebræ will be elucidated in the following case of excurvation of the spine.

CASE IV.

Miss Jane Archer, æt. seven, (56, Wild-street,) born of parents free from scrofula. Her father is subject to asthma. She is of a thin delicate form, but has no strumous appearance. She has been affected with excurvation of the dorsal vertebræ during the last five years and a half, which has gradually formed, but has increased very much since Christmas last to this period, (May 29th,) which rapid increase is attributed by the parents to another fall she received at that time, but is really owing to the principle above mentioned. The curvature embraces the twelve dorsal vertebræ, and forms a regular arc, whose chord

* There is an excellent specimen of this in Mr. Brookes's museum.

line measures only four inches, three and a half lines!* The spine, of course, forms a hump projecting behind, the scapulæ appear to be placed on the sides, the ribs are "flattened on their sides,"—that is, they do not describe their usual curve, and are elongated; the sternum projects, and is much raised and depressed by respiration. The lower portion of the second division of the sternum projects to a point, and the cartilago ensiformis is turned inwards, so that the whole sternum presents the form of a broken bow, as if broken at the point of union of the second division of the sternum with the ziphoid cartilage. She has not been entirely paralytic, but has been very weak, and unable to walk without placing her hands on her knees to support her body, and remove the weight from the spinal column. Her parents inform me, her muscular strength was much restored by the use of the vapour-bath three times a-week. She is still weak and thin, and her rest is interrupted by a pain about the posterior superior spinous process of the ileum. She is affected with dyspnœa and cough, and once suffered an attack of asthma. Her digestive organs perform their functions well, and she has no tightness or stricture across the epigastrium, usual in excurvations. On May 29th, 1822, after extending the spine and pressing gently on the projection, the compress, pad, and bandage were applied around the chest, without a shield, and the bandage was fixed by shoulder-straps. The apparatus and extension were renewed every other day. Extension was frequently employed by the parents. The patient observed the facial horizontal position.

*June 2d.*—The measurement of the different derangements of the chest and spine was taken. The greatest projection of the central spinous process of the displaced dorsal vertebræ beyond the spinal line of the lumbar, is three inches. The greatest projection of the same process beyond the spinal line of the last cervical, is one inch nine lines. The whole of the dorsal vertebræ constitute the segment of a circle, nearly equal to a semicircle. The projection of the most protruding dorsal vertebra beyond the twelfth, is two and a half inches. The breadth of the chest from side to side, is six inches. Depth of the chest from the extreme sternal projection to the extreme dorsal, is eight and a half inches. Length of the body, is three feet two inches. The head falls in between the shoulders.

*June 4th.*—Three spinous processes are nearly reduced to a straight line, and the sternum projects less. Used the vapour-bath.

* The natural length of the twelve dorsal vertebræ in a girl of her age should be about seven or eight inches: it was hence very probable that some of the bodies of the vertebræ were wholly destroyed, and that a perfect recovery could not be promised.

*June* 9*th.*—Four spinous processes nearly reduced to a level. Measured again. The greatest projection of the dorsal vertebræ beyond the axis or spinal line of the lumbar, two inches one quarter. The greatest projection of the same beyond the twelfth dorsal, is one inch seven lines. Breadth of the chest, is six and a half inches. Depth of the chest, seven and a half inches. She derives much support from the apparatus, and feels as "if she were going to pieces" when they are taken off for a short time.

*June* 15*th.*—The general health has been good, and the patient went into the country for a few days.

*June* 29*th.*—The general health is still good. Four spinous processes are on a level. The greatest projection of the dorsal vertebræ beyond the spinal line of the lumbar, is reduced to one inch seven lines. The greatest projection of the same beyond the spinal line of the seventh cervical, is only one inch five lines. Breadth of chest, seven inches one line. Depth of chest, seven inches five lines. Length of body, three feet two and a half inches.

*July* 15*th.*—The general health is still good, although she has slight dyspnœa. The greatest projection of the dorsal vertebræ beyond the spinal canal of the first lumbar, is one inch two and a half lines. The greatest projection of the same beyond the spinal line of the seventh cervical, is one inch five lines. Depth of chest, seven inches three lines; breadth, seven inches. Length of body, three feet three inches, which was not increased during her life.

*August* 4*th.*—The spinous processes of the eleventh and twelfth dorsal vertebræ have regained their natural situation. The greatest projection of the most prominent spinous process beyond that of the twelfth dorsal, is one inch. The greatest projection of the same beyond the spinal line of the seventh cervical, is one inch three lines.

*August* 31*st.*—The greatest projection of the most prominent spinous processes of the dorsal vertebræ, beyond that of the eleventh, now in situ, only seven lines. The greatest projection beyond that of the seventh cervical, one inch five lines. The length of the chord line of the arc, including the eleventh and twelfth dorsal, is now five inches one quarter. Depth of chest, seven inches; breadth of chest, seven inches five lines.

During this period the sternum and anterior part of the chest had in a great degree recovered their natural shape, and the patient was no longer chicken-breasted. The ribs on the sides were also much more curved. The pain on the posterior part of the ilium soon disappeared, and did not return. The general health was very good, and she had improved in every respect, agreeably to the testimony of all. She paid a visit of some days to the country; and on her return, at the end of September, she was seized with a most untractable attack of asthma, of which she died early in October. Her father had firmness and philosophy enough to allow an examination after death, which presented the following appearances:

The muscles of the back appeared to be stretched where they passed around the greatest convexity of the excurvation, and their volume in this situation was much diminished. The inter-spinous ligaments were tense. We took the liberty of sawing out, and bringing away, the greatest portion of the spine, with parts of the posterior ends of the ribs attached to it. On removing the spine, the lungs presented a mass more like dark coagulated blood than the parenchyma of the lungs, so much were they gorged. They adhered pretty generally to the costal pleura. The bodies of the seventh, eighth, ninth, tenth, and eleventh vertebræ, were entirely removed by absorption, and the superior and anterior portions of the body of the twelfth dorsal were also absorbed; while their processes remained, although altered in structure and curved. The seventh, eighth, ninth, tenth, and eleventh spinous processes are united by anchylosis. The posterior ends of the ribs are close to each other, and are attached to the anterior part of the transverse processes. The inferior part of the seventh rib is partly destroyed by the absorbents. The ligamentum anticum commune is in the same tense state as in health, except where the bodies of the vertebræ are destroyed: in this situation it is thickened and loose, and covers a small ætheromatous tumor, which it surprised us to find thus situated. Nature had begun to deposit ossific matter on the vertebral side of this ligament, and it was covered with fat towards the abdomen. The engraving will convey a correct idea of the curvature* of the ten dorsal vertebræ removed; the extent of the outer line of curve being four inches seven lines; of the inner line, one inch three lines. The distance between the sixth dorsal vertebra and the remains of the twelfth dorsal, is only four lines!

The spinal canal and vertebræ were sawn through, in the presence of my friend Mr. Copland Hutchison, and a professor of surgery from Berlin. The spinal canal and medulla canalis were preserved continuous, although both, in some parts, had their natural dimensions diminished, and both deviated from the natural spinal course, by being forced to take a circuitous one around the curvature. The spinal marrow and its membranes were merely covered by the spinous processes where the excurvation is most prominent and convex. These processes

* While living, the muscles prevented the upper dorsal vertebræ from inclining so much forwards.

do not form a thick defence, as, in one or two parts, they are not perfectly anchylosed, or united by bone. The diameter of the spinal canal in the dorsal vertebræ above where it is contracted, is six lines. The diameter, in its narrowest or contracted part, is four lines. The diameter of the canal in the lumbar vertebræ below the contracted part, is seven lines. The foramina for the passage of the nerves remain entire, although some appear diminished in size. On examining the bodies of the vertebræ after they were divided by the saw, we all agreed there was not the slightest appearance of caries, and that the bodies of the vertebræ had, in all probability, been destroyed by progressive absorption.—This specimen of diseased spine will be found in Mr. Brookes's museum.

[To be continued.]

*37, Bedford-street, Covent Garden;*

*November 26th,* 1822.

## Figures and Tables

**Figure f1:**